# Full-Spectrum Surveillance of Pre-Treatment HIV Drug Resistance in Southeastern China

**DOI:** 10.3390/ph17070900

**Published:** 2024-07-06

**Authors:** Jiafeng Zhang, Baochang Sun, Zihang Sheng, Xiaobei Ding, Qin Fan, Gang Huang, Zhihong Guo, Ping Zhong, Lingjie Liao, Hui Xing, Yan Xia, Chengliang Chai, Jianmin Jiang

**Affiliations:** 1 Department of HIV/AIDS Control and Prevention, Zhejiang Provincial Center for Disease Control and Prevention, Hangzhou 310051, China; jfzhang@cdc.zj.cn (J.Z.); xbding@cdc.zj.cn (X.D.); qfan@cdc.zj.cn (Q.F.); 15058462872@163.com (G.H.); zhhguo@cdc.zj.cn (Z.G.); yanxia@cdc.zj.cn (Y.X.); 2 Department of Microbiological Test, Wenzhou Municipal Center for Disease Control and Prevention, Wenzhou 325001, China; sbch1532@126.com; 3 School of Laboratory Medicine and School of Life Sciences, Wenzhou Medical University, Wenzhou 325035, China; sfhszh0127@126.com; 4 Health Science Center, Ningbo University, Ningbo 315211, China; 5 Shanghai Municipal Center for Diseases Control and Prevention, Shanghai 200336, China; zhongp56@163.com; 6 Division of Virology and Immunology, National Center for AIDS/STD Control and Prevention (NCAIDS), Beijing 102206, China; liaolj@chinaaids.cn (L.L.); xingh@chinaaids.cn (H.X.)

**Keywords:** HIV-1, pre-treatment drug resistance, nucleoside reverse transcriptase inhibitor, non-nucleoside reverse transcriptase inhibitor, protease inhibitor, integrase strand transfer inhibitor

## Abstract

HIV drug resistance compromises the ability of anti-retroviral therapy (ART) to suppress viral replication, resulting in treatment failure. This study investigates the prevalence of pre-treatment drug resistance (PDR) in newly diagnosed individuals in a prosperous city (Wenzhou) in Southeastern China. A cross-sectional investigation was carried out among 473 newly diagnosed ART-naive HIV-1-infected individuals between January and December 2022. The protease–reverse transcriptase (PR-RT) region and integrase (IN) region of HIV-1 were amplified by two separately nested PCRs, followed by sequencing. Drug resistance mutations (DRMs) and drug resistance to nucleoside reverse transcriptase inhibitors (NRTIs), non-nucleoside reverse transcriptase inhibitors (NNRTIs), protease inhibitors (PIs) and integrase strand transfer inhibitors (INSTIs) were analyzed. The PDR prevalence was 6.5% [95% CI: 4.4–9.1] for any anti-retroviral drug, 0.9% [95% CI: 0.3–2.3] for NRTIs, 4.1% [95% CI: 2.5–6.5] for NNRTIs, 1.8% [95% CI: 0.8–3.6] for PIs and 0.5% [95% CI: 0.1–1.8] for INSTIs. According to the subtyping results of the PR-RT region, 11 different subtypes and 31 unique recombinant forms (URFs) were found. CRF07_BC was the dominant subtype (53.7%, 233/434), followed by CRF01_AE (25.3%, 110/434). V179D (1.6%) and K103N (1.4%) were the most predominant types of NNRTI DRMs. Q58E (1.2%) and M184V (0.7%) were the most frequent PI DRMs and NRTI DRMs, respectively. The INSTI-related DRMs Y143S (causes high-level resistance to RAL) and G163K (causes low-level resistance to EVG and RAL) were found in one patient each. Given the relatively high PDR prevalence of NNRTI (4.1%), non-NNRTI-based ART may be preferred in the future. It is recommended to include genotypic resistance testing before starting ART in regions where feasible.

## 1. Introduction

HAART (high active anti-retroviral therapy) is a main stay of treatment for HIV-infected individuals and AIDS patients (HIV/AIDS), contributing to the remarkable decrease in the morbidity and mortality of HIV/AIDS as well as the further risk of HIV transmission [1,2]. By the end of 2022, 29.8 million people were receiving ART globally [3], and among these individuals, approximately 1.1 million people with HIV/AIDS were living in China. Pre-exposure prophylaxis (PrEP) and post-exposure prophylaxis (PEP) are also becoming more prevalent in high-risk groups (e.g., men who have sex with men) in China [4]. It is worth noting that access to ART has been increasingly diversified in developed regions in China, including free ART under the “Four Frees and One Care” policy and self-pay for second- or third-line drugs.

In China, the national free ART program was initiated in 2003 and rapidly expanded nationwide. Currently, the first-line ART regimens in China consist of two NRTIs and one NNRTI. In general, the increased use of ART regimens has been accompanied by the emergence of drug resistance, which leads to treatment failure and transmission to new infections [5,6,7]. There are growing concerns over the increase in pre-treatment drug resistance (PDR) in China. However, it is inevitable due to the rapid and error-prone replication of HIV, a high mutation rate under drug-selective pressure and persistent HIV transmission in the population [8]. According to the World Health Organization (WHO) HIV drug resistance report 2021, the prevalence of PDR to efavirenz or nevirapine among adults initiating ART exceeded 10% for 70% (21/30) of the countries reporting data to the WHO between 2014 and 2020 [9]. A systematic review spanning 2001–2017 indicated that the pooled prevalence of transmitted drug resistance (TDR) in China was 3.0% (95% CI: 2.8–3.2%) [10]. According to a nationwide study conducted in 2017 from 13 provinces or cities, the overall PDR prevalence was 6.8% in China [11]. The overall prevalence of PDR was 7.4% (190/2568) according to a national study based on eight provinces in China in 2022 [12]. However, TDR or PDR prevalence show wide regional differences. Some parts of China show worrying signs of pre-treatment resistance, for example, the prevalence of pre-treatment resistance was 18.3% in Xi’an [7], 13.5% in Tianjin [6] and 17.4% in Shanghai [13].

Integrase strand transfer inhibitors (INSTIs) are a new class of drugs that target HIV integrase, offering patients novel options for treatment. One INSTI plus two NRTIs are recommended regimens for ART-naive patients by guidelines for the treatment of HIV/AIDS in Europe [14]. INSTI-based regimens are recommended by the International Antiviral Society—USA (IAS-USA) for most individuals owing to their high efficacy, tolerability, safety, and high barrier to resistance [15]. However, INSTIs do not account for a high proportion of ART regimens in China. Drug resistance mutations (DRMs) may occur despite the high efficacy of INSTIs as anti-retroviral drugs against HIV infection. In previously published studies, major INSTI resistance mutations were identified in newly diagnosed HIV-1 patients in Europe and the United States [16,17]. In general, published data regarding PDR in China are incomprehensive and typically do not include INSTIs. Previous studies reported major INSTI resistance mutations among treatment-naive patients in Guangdong [18], Jiangsu [19] and Henan [20]. PDR prevalence displayed spatial heterogeneity across different areas because of unsynchronized economic development and differentiated antiviral treatment regimens. Surveillance of HIV PDR in economically active areas is necessary and can provide important data for understanding the full picture of the country.

Wenzhou, situated on the southeastern coast of China, is a prosperous city with a dynamic economy, bustling trade, and entrepreneurial spirit. According to statistics in 2022, the region has a permanent population of 9.6 million, among which 4.1 million are migrants (42.7%). The cumulative number of HIV/AIDS cases was 5769 in Wenzhou by the end of 2022, with 585 new cases identified in the year 2022. The majority of patients (>90%) with ART in the region are on first-line regimens (NRTIs/NNRTIs), while the proportion of second-line regimens with PIs or INSTIs regimens is estimated to be 5–10%, which shows an increasing trend. The region is at risk of a rising HIV epidemic as well as an increased risk of transmitted drug resistance due to diversified ART drug use and frequent population movements. In the present study, we conducted a comprehensive investigation of HIV PDR (including NRTIs, NNRTIs, PIs and INSTIs) among ART-naive patients in Wenzhou. Our study provides a wealth of PDR surveillance data to benefit the development of preventive HIV/AIDS control strategies.

## 2. Results

### 2.1. Demographic Characteristics of the Study Subjects

A total of 473 newly confirmed HIV-positive individuals in Wenzhou in 2022 were enrolled in this study (Table 1). The majority of the subjects were male (82.0%, 388/473), and females accounted for 18.0% (85/473). The median age of these individuals was 44 years (range 16–87 years). The distribution of marital status was as follows: 151 single (31.9%); 193 married (40.8%); 120 divorced or widowed (25.4%); and 9 unknown (1.9%). The predominant subjects (67.9%) had a junior high school education or lower. In terms of occupation distribution, domestic/housework workers and unemployed individuals accounted for 35.7% of the subjects (169/473), followed by workers (18.6%), commercial service workers (18.6%), peasants (12.9%) and other occupations accounting for 14.2% (67/473). The mode of transmission was mainly heterosexual transmission (60.9%, 288/473), followed by homosexual transmission (36.6%, 173/473).

### 2.2. Distribution of HIV-1 Subtypes

In all, 434 (434/473, 91.8%) sequences of the PR-RT region were obtained, while 407 (407/473, 86.0%) sequences of the IN region were acquired for further analysis. According to the subtyping results of the PR-RT region, CRF07_BC was the dominant subtype (53.7%, 233/434), followed by CRF01_AE(25.3%, 110/434), C(3.7%), CRF08_BC(3.7%), B(2.5%), CRF55_01B(2.1%), CRF85_BC(0.7%), CRF59_01B(0.5%), CRF02_AG(0.2%), CRF67_01B(0.2%), CRF68_01B(0.2%), URF(CRF01_AE/07_BC)(3.9%), URF(B/C)(2.1%), URF(CRF01_AE/BC)(0.5%), URF(CRF01_AE/C)(0.5%) and URF(CRF07_BC/C)(0.2%).

### 2.3. PDR Prevalence

In the univariate analysis for identifying risk factors associated with PDR, no statistically significant differences were observed for categorical variables, including gender, age group, marital status, occupation, transmission route and first CD4 count after HIV confirmation (all *p* > 0.05) (Table 1).

The overall prevalence of PDR in the participants was 6.5% (30/464) [95% CI: 4.4–9.1]. Specifically, the PDR prevalence was 0.9% (4/434) [95% CI: 0.3–2.3] for NRTIs, 4.1% (18/434) [95% CI: 2.5–6.5] for NNRTIs and 1.8% (8/434) [95% CI: 0.8–3.6] for PIs. Two INSTI-related major DRMs were detected among the enrolled individuals with qualified sequences (n = 407), representing a PDR prevalence of 0.5% (2/407) [95% CI: 0.1–1.8] for INSTIs.

**Figure 1:**
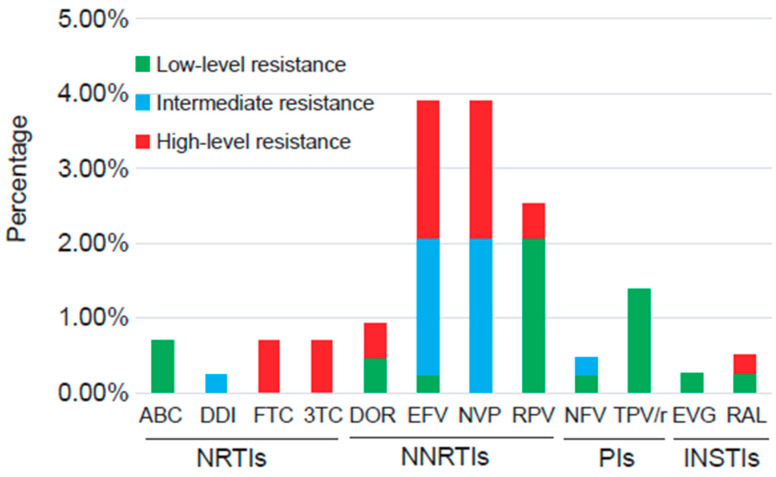
Percentages of drug resistance to different ART drugs among HIV-1-infected individuals in Wenzhou, China, interpreted by the Stanford HIV Drug Resistance Database.

The resistance frequency for specific drugs is shown in Figure 1. Four participants showed drug resistance to NRTIs. Three participants (0.7%, 3/434) showed low-level resistance to ABC and high-level resistance to FTC and 3TC. The remaining participant showed intermediate-level resistance to DDI. For NNRTIs, EFV and NVP both displayed the highest resistance frequency (3.9%, 17/434), followed by RPV (2.5%, 11/434) and DOR (0.9%, 4/434). Eight participants showed resistance to PIs, including six (1.4%) participants who were low-level resistant to TPV/r and two (0.5%) participants who were resistant to NFV. Two participants showed resistance to INSTIs. One participant showed low-level resistance to EVG and RAL, and the other showed high-level resistance to RAL.

### 2.4. Drug Resistance Mutations’ Distribution

A total of 32 DRMs that cause low-level and above resistance were identified in 30 individuals diagnosed with PDR (Table 2). Among these 30 individuals, the largest proportion were aged between 25 and 49 years (46.7%). Domestic workers and unemployed individuals were the most frequent occupations (36.7%), followed by peasants (23.3%). In terms of marital status, married individuals were the majority (53.3%). The predominant subtypes were CRF01_AE (43.3%, 13/30) and CRF07_BC (40.0%, 12/30). The majority of the individuals (73.3%) had their first CD4 lymphocyte counts lower than 350 cells/μL after HIV confirmation, indicating a high proportion of late detection.

**Figure 2:**
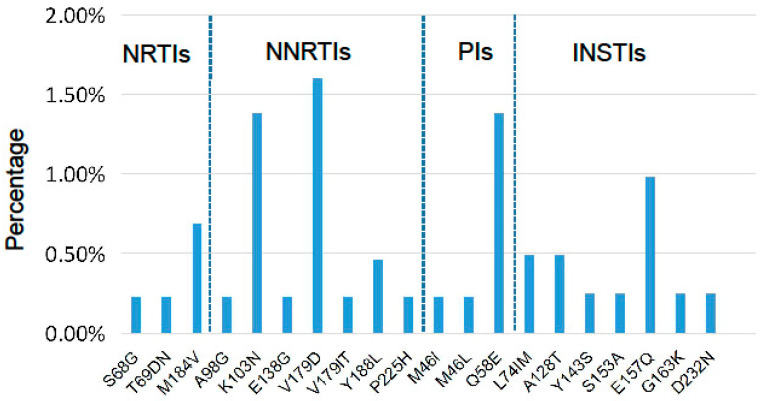
Percentages of HIV-1 pre-treatment drug resistance (PDR) mutations to NRTIs, NNRTIs, PIs and INSTIs among HIV-1-infected individuals in Wenzhou, China.

The DRM distribution is depicted in Figure 2. Among NRTI-related DRMs, M184V (0.7%), S68G (0.2%) and T69DN (0.2%) were identified. M184V (0.7%) was the most frequent NRTI DRM. Among NNRTI-related DRMs, V179D (1.6%), K103N (1.4%), Y188L (0.5%), A98G (0.2%), E138G (0.2%), V179IT (0.2%) and P225H (0.2%) were found. V179D (1.6%) and K103N (1.4%) were the most predominant types of NNRTI DRMs. Among PI-related DRMs, Q58E (1.4%) and M46L (0.2%) were detected. Q58E (1.4%) was the most predominant PI DRM.

Most of the individuals (93.3%, 28/30) with PDR harbored a single-class mutation. Two individuals carried two classes of resistance mutations—specifically, patient WZ2922002 with DRMs of M184V (NRTI) + K103N (NNRTI) and patient WZ0122082 with DRMs of K103N (NNRTI) + Q58E (PI). The former DRMs cause high-level resistance to FTC, 3TC, EFV and NVP and low-level resistance to ABC. The latter DRMs cause high-level resistance to EFV and NVP and low-level resistance to TPV/r.

A total of 13 individuals harbored INSTI-related DRMs, among which the major DRMs Y143S (causes high-level resistance to RAL) and G163K (low-level resistance to EVG and RAL) were found in one patient each. Eleven individuals harbored only INSTI accessory DRMs, among which E157Q (1.0%), L74IM (0.5%), A128T (0.5%), S153A (0.2%), G163K (0.2%) and D232N (0.2%) were found.

## 3. Discussion

In the present study, we conducted a comprehensive PDR survey among newly diagnosed HIV-infected individuals in a prosperous city (Wenzhou) in Southeastern China. The study covered all four categories of ART drugs currently available, namely NRTIs, NNRTIs, PIs and INSTIs. Heterogeneity in PDR in the different categories of ART drugs was observed, highlighting the current situation of antiviral drugs and the direction of future optimization.

The prevalence of PDR to NRTIs, NNRTIs, PIs and INSTIs was 0.9% [95% CI: 0.3–2.3], 4.1% [95% CI: 2.5–6.5], 1.8% [95% CI: 0.8–3.6] and 0.5% [95% CI: 0.1–1.8], respectively. The prevalence of PDR to NNRTIs was relatively high, and these drugs have been widely used and for long terms in first-line ART regimens in China since 2003. The PDR to NNRTIs (4.1%) in this study was comparable to those in Nanjing (4.8%) [21] and fell within the range (3.7~10.3%) of the national PDR surveillance in China in 2022 [12], but lower than those in recent studies in Shanghai (16.4%) [13], Tianjin (12.5%) [6] and Xi’an (16.1%) [7]. Given the relatively high PDR prevalence of NNRTI, non-NNRTI-based ART may be preferred in the future. The prevalence of PDR to PIs (1.8%) in the current study was similar to or slightly higher than that reported in the above regions, where it was mostly less than 1.0%. This could reflect a more extensive use of PIs in Wenzhou than in some first-tier cities in China. Wenzhou is one of the most active regions in China’s private economy and private capital. The proportion of self-funded ART drugs in Wenzhou is at the forefront of regions in Zhejiang Province. Therefore, our findings remind us of the necessity for continued surveillance of transmitted drug resistance in Wenzhou. The prevalence of PDR to INSTIs was as low as 0.5%, suggesting that there are still ample choices for replacing new drugs if there is resistance to first-line drugs. Given that PIs and INSTIs are not widely used in China, the lower resistance rate is understandable.

The most common NNRTI-associated mutation in this study was V179D, which was similar to previous reports [7,13]. V179D can cause intermediate-level resistance to EFV and NVP and low-level resistance to RPV. In combination with other NNRTI DRMs, it appears to contribute low levels of reduced susceptibility to each of the NNRTIs [22]. We found that six patients harbored the PI accessory mutation Q58E, which can cause low-level resistance to TPV [22]. As the most commonly occurring NRTI DRM [23], M184V was detected in three patients in this study, and it confers high-level resistance to FTC and 3TC and low-level resistance to ABC [22]. As a novel class of ART drugs, INSTIs show high antiviral potency and high-resistance barriers. However, we found a major DRM, Y143S, which causes high-level resistance to RAL, highlighting a challenge faced with rapid and unexpected changes. One patient with the G163K mutation exhibited low-level resistance to EVG and RAL. We observed that four individuals (1.0%) harbored the accessory INSTI resistance mutation E157Q. This mutation is a polymorphic mutation selected in persons receiving RAL and EVG and appears to have little effect on INSTI susceptibility [24]. Due to the booming economy, the proportion of INSTIs used in the ART population in the form of self-paid medication in Wenzhou reached more than 20%, which is much higher than most areas in Zhejiang Province. We should still be cautious in our optimism of the PDR in Wenzhou, however. PDR surveillance should be continually performed to improve treatment strategies and public health responses.

Pre-exposure prophylaxis (PrEP) and post-exposure prophylaxis (PEP) are important tools to prevent HIV transmission and have been increasingly used in high-risk populations in recent years in China [4]. It has been reported that the risk of resistance may be increased in individuals receiving PrEP around the time of HIV infection and in those with prolonged PrEP use after HIV infection [25,26]. When unaware of their HIV infection, some individuals may continue to take PrEP or PEP regimens. Delaying the ART switch may promote drug resistance [5]. The WHO recommends that PrEP scale-up be accompanied by the surveillance of HIV drug resistance [9]. Therefore, there is no unequivocal answer on this matter, and it warrants continued attention.

In this study, we did not carry out further epidemiological investigations for individuals with PDR, making it difficult to trace the source of the resistant strains. As the most common approach for HIV drug resistance testing, we used Sanger sequencing to detect variants with a frequency of more than 20% of the total quasispecies pool [27]. Hence, most minority strains (<20%) will not be detected in this study, which will underestimate the prevalence of DRMs. Future application of next-generation sequencing (NGS) can effectively detect low-frequency drug-resistant strains, which is expected to overcome this deficiency. Logistic regression analysis did not identify any statistically significant differences, which may be attributed to the sample size. Further investigation is required to identify the risk factors for PDR.

## 4. Materials and Methods

### 4.1. Study Population and Sample Collection

We carried out a cross-sectional and analytical study throughout 2022 in Wenzhou in Zhejiang Province in Southeastern China. Patient recruitment and sample collection were conducted by the Wenzhou Municipal Center for Disease Control and Prevention in collaboration with 12 county/district centers for disease control and prevention within their jurisdiction. The inclusion criteria of participants were as follows: (1) newly confirmed HIV-positive in Wenzhou; (2) follow-up and provided written informed consent; (3) had not received any previous antiviral treatment; and (4) eligible whole blood with ethylenediaminetetraacetic acid (EDTA) was collected. A total of 473 participants and eligible samples were included in this study. Plasma was separated from whole blood by centrifugation at 3000× *g* for 10 min and then stored at −80 °C until further use. This study was approved by the Medical Ethics Committee of the National Center for AIDS/STD Control and Prevention. In this study, all methods were performed according to approved guidelines and regulations. Sociodemographic data for the participants (including age, gender, transmission route, occupation, marital status, etc.) were extracted from the Chinese HIV/AIDS Comprehensive Response Information Management System (CRIMS).

### 4.2. Nucleic Acid Extraction and Amplification

Total viral RNA was extracted from plasma using a QIAamp 96 Virus QIAcube HT Kit (Qiagen, Hilden, Germany) according to the manufacturer’s instructions. Reverse transcriptase–polymerase chain reaction (RT-PCR) and nested PCR were used to amplify two *pol* fragments, namely, the protease–reverse transcriptase (PR-RT) region (encoding the protease and the first 299 residues of the reverse transcriptase gene) and the integrase (IN) region (covering all 288 amino acids of integrase). The lengths of the two amplified fragments were 1316 bp (HXB2: 2147–3462) and 948 bp (HXB2: 4141–5088). PrimeScript^TM^ One Step RT-PCR Kit Ver. 2 (Takara, Dalian, China) and the Ex *Taq* Kit (Takara, Dalian, China) were applied in the amplification. The primer sequences and thermal cycling conditions for the PR-RT region were described previously [28]. Amplification of the entire IN gene was performed as previously described [29]. Positive PCR products were separated by 1% agarose gel electrophoresis and sent to Hangzhou TsingKe Bio-Tech Co. for purification and Sanger sequencing with an ABI 3730XL DNA sequencer (Applied Biosystems, Carlsbad, CA, USA) using overlapping sequencing primers.

### 4.3. Subtyping and Genotypic Drug Resistance Analysis

The obtained sequences were trimmed, assembled and adjusted with Sequencher v5.4.6 (Genecodes, Ann Arbor, MI, USA). Then, the sequences were aligned using BioEdit v7.2.0. The subtypes were determined using the online tool COMET HIV-1 (https://comet.lih.lu/index.php?cat=hiv1, accessed on 30 June 2024) and then verified by neighbor-joining (NJ) phylogenetic tree analysis using MEGA v6.0 (Kimura two-parameter model with 1000 bootstrap replicates).

Genotypic drug resistance analysis was performed by HIVdb Program v9.4.1, which integrates with the Stanford University HIV Drug Resistance Database (https://hivdb.stanford.edu/, accessed on 30 June 2024). The HIVdb can identify DRMs and infer levels of resistance to the most common ART drugs. We tested a total of 25 commonly utilized ART drugs, namely abacavir (ABC), zidovudine (AZT), emtricitabine (FTC), lamivudine (3TC), tenofovir disoproxil fumarate (TDF), stavudine (D4T) and didanosine (DDI) for NRTIs; doravirine (DOR), efavirenz (EFV), etravirine (ETR), nevirapine (NVP) and rilpivirine (RPV) for NNRTIs; atazanavir/ritonavir (ATV/r), darunavir/ritonavir (DRV/r), lopinavir/ritonavir (LPV/r), fosamprenavir/ritonavir (FPV/r), indinavir/ritonavir (IDV/r), nelfinavir (NFV), saquinavir (SQV) and tipranavir (TPV) for PIs; and bictegravir (BIC), cabotegravir (CAB), dolutegravir (DTG), elvitegravir (EVG) and raltegravir (RAL) for INSTIs. Sequences associated with low-level, intermediate or high-level resistance were defined as conferring drug resistance.

### 4.4. Statistical Analysis

All statistical analyses were performed using IBM SPSS V19.0 (IBM, Armonk, NY, USA). Variables were summarized as medians (ranges) for continuous variables and numbers (%) for categorical variables. Logistic regression analysis was performed to identify potential risk factors associated with PDR. A *p* value < 0.05 was required for a variable to remain in the further adjustment. First, crude odds ratio (OR) with the corresponding 95% confidence intervals (CIs) were calculated to show the strength of the associations. We then performed multivariable analyses to calculate an adjusted odds ratio (AOR) and 95% CI.

## 5. Conclusions

The overall prevalence of PDR in Wenzhou has not reach the 10% threshold, which suggests that ART drugs currently have good applicability overall and that the use of PIs and INSTIs is expected to bring ideal viral suppression. A total of 32 major mutations were detected in 30 HIV-1 patients from Wenzhou, emphasizing the importance of PDR monitoring before initiating ART. We also propose the incorporation of pre-ART genotypic resistance testing into clinical care for better treatment outcomes in regions where feasible.

## Figures and Tables

**Table 1:** Demographic characteristics of study patients and factors associated with PDR.

Variables	Subjects (N = 473)	PDR	Crude OR ^a^ (95% CI ^b^)	*p*
**Gender**				
Male	388 (82.0)	27	1	
Female	85 (18.0)	3	0.49 (0.15–1.66)	0.253
**Age group (years)**				
<25	58 (12.3)	3	1	
25–49	226 (47.8)	14	1.19 (0.33–4.29)	0.791
50–59	102 (21.6)	6	1.13 (0.27–4.69)	0.869
≥60	87 (18.4)	7	1.57 (0.39–6.33)	0.529
**Marital status**				
Single	151 (31.9)	8	1	
Married	193 (40.8)	16	0.62 (0.26–1.49)	0.282
Divorced/widowed	120 (25.4)	4	0.38 (0.12–1.16)	0.090
Unknown	9 (1.9)	2	3.09 (0.59–16.13)	0.181
**Education**				
Primary school or illiterate	154 (32.6)	8	1	
Junior high school	167 (35.3)	14	1.66 (0.68–4.07)	0.271
Senior high school	77 (16.3)	6	1.50 (0.50–4.49)	0.468
College or above	75 (15.9)	2	0.50 (0.10–2.42)	0.389
**Occupation**				
Workers	88 (18.6)	4	1	
Peasants	61 (12.9)	7	2.68 (0.75–9.58)	0.131
Commercial service workers	88 (18.6)	6	1.52 (0.41–5.59)	0.529
Domestic workers and unemployed individuals	169 (35.7)	10	1.30 (0.40–4.27)	0.667
Cadres, staff, students, teachers and doctors	35 (7.4)	2	1.23 (0.21–7.03)	0.818
Other and unknown	32 (6.8)	1	0.65 (0.07–6.08)	0.653
**Transmission route**				
Homosexual behavior	173 (36.6)	13	1	
Heterosexual behavior	288 (60.9)	17	0.77 (0.36–1.62)	0.487
Other	12 (2.5)			
**First CD4 count after HIV confirmation (cells/µL)**				
<200	189 (40.0)	13	1	
200–499	233 (49.3)	14	0.88 (0.40–1.92)	0.750
≥500	45 (9.5)	2	0.67 (0.15–3.10)	0.612
Missing	6 (1.3)	1	2.69 (0.29–24.78)	0.382

^a^ OR: odds ratio, ^b^ CI: confidence interval.

**Table 2:** Characteristics of patients with DRMs.

Sample ID	Gender	Age (Years)	Transmission Route	Occupation Type	Marital Status	HIV Subtypes	CD4 Count (Cells/µL)	DRMs ^c^			
								NRTI	NNRTI	PI	INSTI
WZ0122052	Female	40	HET ^a^	Domestic workers and unemployed individuals	Married	CRF01_AE	22	S68G, T69DN	-	-	-
WZ2922002	Male	29	HET	Peasant	Unknown	CRF07_BC	285	M184V	K103N	-	-
WZ0122063	Male	37	HET	Domestic workers and unemployed individuals	Single	CRF07_BC	215	M184V	-	-	-
WZ0122118	Male	58	HET	Domestic workers and unemployed individuals	Married	CRF07_BC	119	M184V	-	-	-
WZ8122013	Male	46	HET	Workers	Divorced/widowed	CRF01_AE	240	-	V179D	-	-
WZ0122011	Male	20	HOM ^b^	Workers	Single	CRF01_AE	312	-	Y188L	-	-
WZ0122014	Male	72	HOM	Peasants	Married	CRF01_AE	400	-	V179D	-	-
WZ0122043	Female	29	HET	Domestic workers and unemployed individuals	Married	URF(CRF01_AE/07BC)	42	-	K103N	-	-
WZ0122061	Male	25	HET	Commercial service workers	Single	CRF01_AE	34	-	E138G, V179IT	-	-
WZ0122065	Male	35	HET	Commercial service workers	Single	CRF07_BC	47	-	P225H	-	ND ^d^
WZ0122080	Male	33	HOM	Cadres	Single	CRF01_AE	313	-	V179D	-	-
WZ0122082	Male	45	HOM	Domestic workers and unemployed individuals	Divorced/widowed	CRF07_BC	160	-	K103N	Q58E	-
WZ0122083	Male	30	HOM	Commercial service workers	Married	CRF01_AE	183	-	V179D	-	-
WZ0122116	Male	46	HET	Fisherman	Married	CRF01_AE	198	-	V179D	-	-
WZ0122126	Male	76	HOM	Peasants	Married	CRF59_01B	115	-	K103N	-	-
WZ8122033	Male	27	HOM	Domestic workers and unemployed individuals	Single	CRF01_AE	818	-	V179D	-	-
WZ2722020	Male	65	HET	Peasants	Married	C	399	-	K103N	-	ND
WZ2422023	Male	66	HOM	Peasants	Married	CRF01_AE	333	-	V179D	-	-
WZ2622013	Male	71	HET	Domestic workers and unemployed individuals	Married	CRF01_AE	679	-	Y188L	-	-
WZ2621109	Male	53	HET	Commercial service workers	Unknown	CRF07_BC	Unknown	-	A98G	-	-
WZ0122260	Female	49	HET	Domestic workers and unemployed individuals	Married	CRF59_01B	46	-	K103N	-	-
WZ2621087	Male	51	HET	Peasants	Married	CRF07_BC	310	-	-	Q58E	-
WZ0122142	Male	19	HOM	Students	Single	CRF07_BC	400	-	-	Q58E	-
WZ8122050	Male	36	HOM	Workers	Married	CRF07_BC	251	-	-	Q58E	-
WZ2722016	Male	22	HOM	Peasants	Single	CRF55_01B	420	-	-	M46L	-
WZ2621104	Male	61	HOM	Workers	Divorced/widowed	CRF01_AE	10	-	-	M46I	-
WZ0122226	Male	70	HET	Domestic workers and unemployed individuals	Married	CRF07_BC	17	-	-	Q58E	-
WZ0122267	Male	59	HOM	Commercial service workers	Married	CRF07_BC	215	-	-	Q58E	-
WZ0122031	Male	58	HET	Domestic workers and unemployed individuals	Married	CRF01_AE	162	ND	ND	ND	Y143S
WZ0122252	Male	50	HET	Commercial service workers	Divorced/widowed	CRF07_BC	427	-	-	-	G163K

^a^ HET: heterosexual transmission, ^b^ HOM: homosexual transmission, ^c^ DRMs: drug resistance mutations, ^d^ ND: not detected (due to failed amplification). A dash indicates that no DRM was detected.

## Data Availability

The study database used and/or analyzed during the current study will be made available from the corresponding author upon reasonable request.
